# Sensorimotor rehabilitation and changes in upper limb function after acute cervical spinal disorders: a retrospective case series (*n* = 14)

**DOI:** 10.3389/fmed.2026.1958604

**Published:** 2026-10-21

**Authors:** Inês Margarida Oliveira Agostinho, Ana Temudo, Sérgio Manuel Oliveira Gonçalves Bacelo, Ricardo Picoito, Rafael Bernardes, Luís Sousa

**Affiliations:** 1 Universidade Catolica Portuguesa, Lisbon, Portugal; 2 Unidade Local de Saude de Lisboa Ocidental, Lisbon, Portugal; 3 Centre for Interdisciplinary Research in Health (CIIS), Faculty of Health Sciences and Nursing (FCSE), Universidade Católica Portuguesa, Lisbon, Portugal; 4 Escola Superior de Enfermagem de Lisboa, Lisbon, Portugal; 5 Nursing Department, Escola Superior de Saúde Atlântica, Barcarena, Portugal; 6 Comprehensive Health Research Centre, Polo de Évora, Universidade de Évora, Évora, Portugal

**Keywords:** case reports, neurological rehabilitation, physical therapy modalities, rehabilitation nursing, upper extremity

## Abstract

**Background:**

Upper limb impairment is a common consequence of acute cervical spinal disorders and can substantially compromise functional independence, highlighting the need for rehabilitation strategies that address sensorimotor and functional impairments.

**Objective:**

To describe changes in upper limb function following a sensorimotor intervention incorporating a viscoelastic product in individuals with acute non-degenerative cervical spinal disorders.

**Design:**

Uncontrolled, before and after, observational retrospective longitudinal case series study with no comparator group, conducted over a 14-month period.

**Setting:**

Surgical inpatient unit.

**Participants:**

Fourteen participants with acute non-degenerative cervical spinal disorders receiving a rehabilitation nursing programme, with heterogeneous underlying diagnoses including disc herniation, fracture, space-occupying lesion, myelopathy, and spinal cord trauma.

**Main measures:**

Spasticity assessed using the Modified Ashworth Scale, upper limb muscle strength assessed using the Medical Research Council Muscle Scale, and functional independence assessed using the Barthel Index.

**Results:**

Statistically significant changes in spasticity were observed between baseline and discharge (*Z* = −3.093; *p* < .001; *r* = 0.83), together with significant changes in muscle strength across all assessed upper extremity segments: fingers (*Z* = −3.330; *p* < .001; *r* = 0.89), wrist (*Z* = −3.324; *p* < .001; *r* = 0.89), forearm (*Z* = −2.953; *p* < .001; *r* = 0.79), elbow (*Z* = −3.084; *p* < .001; *r* = 0.82), and shoulder (*Z* = −2.555; *p* = .008; *r* = 0.68), as well as in functional independence (*Z* = −3.297; *p* < .001; *r* = 0.88).

**Conclusions:**

Changes in spasticity, upper limb muscle strength and functional independence were observed between baseline and discharge. Given the uncontrolled retrospective case-series design and absence of a control or comparison group, these findings should be interpreted with caution, as they cannot be attributed specifically to the intervention and should be considered hypothesis-generating for future research. Further prospective controlled studies are warranted to evaluate the effectiveness of this approach.

## Introduction

Neurological disorders are one of the leading causes of disability worldwide; they can significantly affect upper extremity mobility and, consequently, impair the ability to perform activities of daily living that are essential to people's independence and quality of life (1, 2). In this context, rehabilitation nursing plays a crucial role in promoting functional recovery and restoring independence, making it essential to implement evidence-based and clinically relevant approaches to care delivery (3). Given the constant evolution of knowledge, it is also the responsibility of the Rehabilitation Nurse Specialist to stay up to date and incorporate innovative therapeutic approaches, based on the best available scientific evidence, in order to support functional recovery and optimize participation in daily activities (4).

Conventional rehabilitation nursing practice encompasses a diverse range of clinical interventions, with a focus on promoting mobility, functionality, and independence in individuals participating in a rehabilitation program. These practices include: passive, passive/assisted, active/resisted, and active mobilizations of the upper extremity segments (fingers, wrist, forearm, elbow, and shoulder); self-mobilization; cross-facilitation; sensory stimulation; antispastic positioning; constraint-induced approaches; as well as instruction and training in adaptive strategies across various domains of self-care, and the prevention of complications associated with immobility and motor dysfunction (3).

Thus, this study examines the use of a sensorimotor intervention involving the application of a viscoelastic product, such as slime, in a rehabilitation program for individuals with acute, non-degenerative cervical pathology (5). This product has specific mechanical and sensory properties including its texture, elasticity and resistance and manipulating it provides multisensory stimulation that may engage tactile, proprioceptive, motor and attentional processes. These characteristics may facilitate active engagement in upper limb exercises by combining sensory stimulation with movement and resistance-based practice. The therapeutic use of viscoelastic materials in rehabilitation remains exploratory, and the available evidence is limited and preliminary (6).

As a sensorimotor intervention in the field of motor rehabilitation, manipulation of the viscoelastic material may facilitate the practice of fine motor skills through controlled movements of the fingers and hands, potentially supporting fine motor coordination and tasks requiring precision and manual dexterity, such as handwriting and handling of small objects. In terms of somatosensory stimulation, the resistance offered by the material when pressure is applied provides tactile and proprioceptive input. This resistance, resulting from the product's viscoelastic properties, allows for a nonlinear mechanical response to the application of force, which may provide concurrent sensory and motor input during upper extremity movements. Accordingly, manipulation of the product may provide opportunities for active movement, graded force production and resistance-based motor practice, thereby integrating sensory input with voluntary motor activity during the execution of functional movements (8–10).

The cervical region is one of the areas of the body with the highest density of mechanoreceptors—including muscle spindles and Golgi tendon organs—with afferent connections that are essential for maintaining postural control, head stability, and oculocephalic coordination (11, 12). Clinically, altered cervical afferent input and impaired proprioception may be associated with dizziness, headache, postural instability, impaired balance, and disturbances in gait and head-eye coordination (11, 13). These manifestations may support targeted sensorimotor assessment and rehabilitation alongside conventional management. In cases of cervical pathology, alterations in cervical afferent input may impair central sensorimotor integration, leading to measurable changes in joint position sense, balance and muscle recruitment patterns (12, 14). This dysfunction, which is often assessed using the cervical joint position error test, may persist despite conventional musculoskeletal treatment, supporting the need to consider targeted sensorimotor approaches.

Cervical sensorimotor rehabilitation has been shown to be effective in reducing disability and improving range of motion and proprioception in patients with chronic neck pain; an integrative approach combining musculoskeletal intervention and targeted sensorimotor training is recommended (11, 15). In this context, the use of devices with viscoelastic properties may represent a means of providing peripheral somatosensory stimulation (16). Increasing relevant sensory input has been reported to improve joint position awareness and may reduce motor variability by enriching the sensory information available for central integration (15, 16). Therefore, incorporating a viscoelastic product into a structured cervical rehabilitation program may provide an additional source of tactile and proprioceptive input during active upper limb movement and resistance-based exercises. This approach may support sensorimotor integration and motor practice. However, its specific effects on motor recovery in individuals with acute cervical spinal disorders remain uncertain and warrant further investigation.

The present study is also informed by Afaf Meleis's Transitions Model, allowing upper extremity changes to be framed not only as functional deficits but as experiences of change that involve continuous adaptive processes, in line with the dynamic nature of neurological recovery (17). Within the nursing framework, rehabilitation may involve supporting individuals as they acquire “new knowledge and skills to cope with the changes” in their health and functional status (18). Thus, the model provides a conceptual nursing framework for understanding the individuaĺs adaptation to functional changes and their active involvement in the rehabilitation process, while the potential sensorimotor effects of the viscoelastic intervention are considered separately from this theoretical perspective (17).

Following a review of the existing scientific literature, the research question was formulated as follows: “What changes in upper extremity function are observed following a sensorimotor intervention in individuals with acute, non-degenerative cervical spinal disorders?”

Based on this question, the general objective of this study was established: to describe changes in upper limb function following a sensorimotor intervention incorporating a viscoelastic product in individuals with acute non-degenerative cervical spinal disorders. Thus, specific objectives were defined to describe changes observed during the rehabilitation period:

- To assess changes in the degree of spasticity in the affected upper extremity using the Modified Ashworth Scale between baseline and discharge. The scale ranges from 0 (“No increase in muscle tone”) to 4 (Affected part rigid in flexion or extension) and is usually applied to each segment of the intended limb
- To assess changes in muscle strength in the individual between baseline and discharge using the Medical Research Council Muscle Scale. The score ranges from 0 (“No visible or palpable muscle contraction”) to 5 (“Normal muscle strength”).
- To assess changes in self-care outcomes between baseline and discharge using the Barthel Index. This standardized tool measures patients’ functional independence in basic activities of daily living (ADLs) with scores ranging from 0 to 100, where higher scores indicate greater independence.

The pain variable was not considered, given that other factors influence it, such as subjectivity and prescribed analgesic medication, and its retrospective assessment would not allow the changes observed to be attributed to the sensorimotor intervention.

## Methodology

A retrospective observational, descriptive, longitudinal case series design was employed, as it is well-suited for exploring phenomena that have not yet been rigorously investigated and allows for an in-depth analysis of interventions and their outcomes in a clinical setting (19). This retrospective case series investigated the implementation of a sensorimotor rehabilitation intervention using a viscoelastic product, during the hospitalization of patients with acute, non-degenerative cervical spinal disorders, who were enrolled in a rehabilitation nursing program conducted over 14 months, from January 2025 to March 2026, at a surgical inpatient unit. No control or comparison group was included. Therefore, changes observed between baseline and discharge were interpreted as within-participant changes occurring during the rehabilitation period and were not considered attributable specifically to the sensorimotor intervention. A consecutive sample of eligible participants was included.

Inclusion criteria comprised adults with acute, non-degenerative cervical spinal disorders, reduced upper extremity mobility, currently enrolled in a rehabilitation program, and provision of informed consent for the use of their data for research purposes.

Variables were selected to characterize the study population and to describe changes observed during the rehabilitation period. They were grouped into three categories: sociodemographic, clinical, and contextual variables.

Sociodemographic variables included age, sex, diagnosis, and length of hospital stay.

Clinical variables analyzed included the presence of tactile sensitivity in the affected upper extremity, the degree of spasticity in the affected upper extremity, muscle strength in the affected upper extremity at the finger, wrist, forearm, elbow and shoulder, and the degree of dependence of the individual. Tactile sensitivity of the affected upper extremity was defined as the ability to perceive tactile stimuli and was recorded dichotomously (present or absent). Spasticity of the affected upper extremity was assessed using the Modified Ashworth Scale, which allows for the quantification of increased muscle tone (20). The Modified Ashworth Scale was recorded using the categories 0, 1, 1+, 2, 3 and 4, with 1+ retained as a distinct category. Muscle strength in the finger, wrist, forearm, elbow, and shoulder of the affected upper extremity was assessed using the Medical Research Council (MRC) scale, which classifies muscle strength on an ordinal scale from 0 to 5 (21). The degree of dependence in performing activities of daily living was assessed using the Barthel Index, with higher scores indicating greater independence (22, 23).

Other treatments and rehabilitation interventions received during hospitalization were also recorded, where documented including surgical management, conventional rehabilitation interventions, occupational therapy, splinting or orthotic management, pharmacological treatment, antispastic medication and conventional upper limb exercises. These treatments were considered as co-interventions that could contribute to changes observed between baseline and discharge.

The number of interventions was defined as the total number of documented rehabilitation nursing sessions involving the viscoelastic product during hospitalization. The post-discharge destination corresponded to the location to which the patient was referred after discharge, categorized as home, home care unit, transfer to another hospital, the Alcoitão Rehabilitation Medicine Center, or the National Network of Integrated Continuing Care.

The participants' progress was monitored through a retrospective analysis of electronic medical records, laboratory test results, and nursing and rehabilitation nursing records documenting the participantś clinical and functional status and the implementation of the sensorimotor intervention. All baseline and discharge clinical assessments were performed by the same rehabilitation nurse specialist throughout the study period. Therefore, inter-rater reliability was not applicable. The collected data were organized into a table in an anonymized database using Excel®. Statistical analysis was performed using SPSS® version 29. Participant characteristics and study variables were summarized using descriptive statistics and are presented as frequencies, percentages and cumulative percentages. For descriptive analysis of muscle strength, data from all 20 affected upper extremities were presented to describe the distribution of MRC grades at the limb level. However, the 20 affected upper extremities were not treated as 20 independent observations in the inferential analysis. For inferential analysis, the Wilcoxon signed-rank test was used to compare paired baseline and discharge measurements at the participant level (*n* = 14). To avoid treating bilateral upper extremities from the same participant as independent observations, one MRC value per muscle segment was derived for each participant. For participants with bilateral involvement (*n* = 6), the MRC scores of the two affected upper extremities were averaged to obtain a single participant-level value for each muscle segment. Thus, when the two limb scores differed, the resulting participant-level value could be fractional (e.g., scores of 2 and 3 yielded a value of 2.5). This procedure was used solely to obtain one paired participant-level measure for the inferential analysis, while the original MRC scores were retained for descriptive reporting. A two-sided *p*-value <.05 was considered statistically significant.

Given the heterogeneity of the underlying cervical spinal disorders, including disc herniation, fracture, space occupying lesion, myelopathy and spinal cord trauma, the pooled results should be interpreted with caution, as these conditions may differ substantially in pathophysiology, neurological involvement and expected recovery trajectories. Subgroup analyses according to diagnosis were considered. However, these were not feasible given the small sample size and the limited number of participants within each diagnostic category. Therefore, the analyses were conducted in the overall sample and are intended to describe observed within-participant changes rather than diagnosis-specific effects.

Because this was an uncontrolled retrospective case series, the analyses were intended to describe changes between baseline and discharge rather than estimate treatment effects attributable to the viscoelastic intervention. This study is part of a broader research study and therefore received approval from the Health Ethics Committee No. 2024-122, and all study participants signed an informed, clear, and voluntary consent form in accordance with Regulation No. 15/2013 of the Directorate-General for Health.

### Sensorimotor intervention program

The sensorimotor intervention was conducted using a viscoelastic product, such as slime, whose texture and elasticity provide a wide range of sensory stimuli. Its physical properties allow for various sensory interactions, including cooling and tactile stimulation. The intervention was intended to provide tactile, proprioceptive and motor stimuli through interaction with the viscoelastic material (9, 10). The product used in this study was manufactured by Canal Toys S.A. (product reference SSC148/SKU SSC148). According to the manufactureŕs declaration of conformity, the product complies with applicable European safety requirements. The product is composed of materials considered non-toxic and commonly used in childreńs toys and complies with European safety requirements, bearing the CE marking. It is recommended for children aged 6 years and older and requires adult supervision.

The sensorimotor intervention program was conducted in an inpatient setting during the acute phase. Participants underwent an initial assessment by a nurse specializing in rehabilitation nursing to determine their eligibility and ability to participate safely in the intervention. Upon inclusion, each participant was provided with an individual, personal, and non-transferable viscoelastic product, stored in its own container and kept in the participant's room. The product was intended for individual use and was not shared between participants. Before and after each session, participants were instructed to perform hand hygiene. The participant was positioned in a stable sitting position, with a hard surface in front of them providing support for the upper extremities, to ensure the safe execution of movements.

Regarding the exercises, the participant was instructed to manipulate the viscoelastic product with the affected upper extremity, either independently or with the support of a healthcare professional and/or family member. The exercises included active movements of the fingers (flexion, extension, abduction, and adduction), together with stretching, pressing, and circular manipulation of the material. These activities were performed according to the participant´s functional capacity and tolerance, providing varied tactile and resistive sensory input. The intervention did not follow a fixed number of repetitions; rather, the duration and type of activity were adapted to the participant´s clinical condition, motor capacity and tolerance.

During the process, participants and their families were encouraged to perform the exercises independently, using the unaffected upper extremity for functional support. Family members were instructed to support the participant's active participation and were advised regarding the safe performance of the exercises and situations requiring interruption of the activity (7). The level of assistance provided by a healthcare professional or family member was adapted to the participant's ability to perform the exercises safely and actively.

In this study, the intended minimum duration of viscoelastic product manipulation was 10 min, adjusted to a maximum of 15 min depending on the participant's tolerance. Sessions were intended to be conducted daily, with a target frequency of three times per day, depending on the individual's condition and response. However, actual intervention exposure varied between participants and was dependent on factors including clinical condition, tolerance, length of hospitalization and ability to perform the exercises independently. Documented sessions represented the sessions recorded in the rehabilitation nursing records and therefore may not have captured all instances of intervention exposure, particularly when exercises were performed independently by participants or with family support. Consequently, the prescribed frequency and duration represented the intended intervention protocol, whereas the documented number of sessions represented only the recorded supervised exposure. The number of sessions therefore reflected the participant´s documented exposure to the intervention during hospitalization rather than a fixed intervention dose.

The viscoelastic product was stored in an individual designated container in the participant's room and was not shared between participants. The material was replaced when its physical properties were considered to have deteriorated and no longer provided the intended viscoelastic characteristics. No participants presented with skin wounds or documented allergies that required exclusion or interruption of the intervention. As the intervention was consistently delivered by the same rehabilitation nurse throughout the study period, no additional team training was required.

## Results

The results are presented using graphs and tables to facilitate interpretation of the findings.

During the study period, 22 patients were assessed for eligibility. Of these, 14 participants met the predefined inclusion criteria and were included in the case series, whereas eight patients were excluded because they had neurological pathology of cerebral origin and therefore did not meet the criterion of an acute non-degenerative cervical spinal disorder. The participant flow is presented in Figure 1.

**Figure 1:**
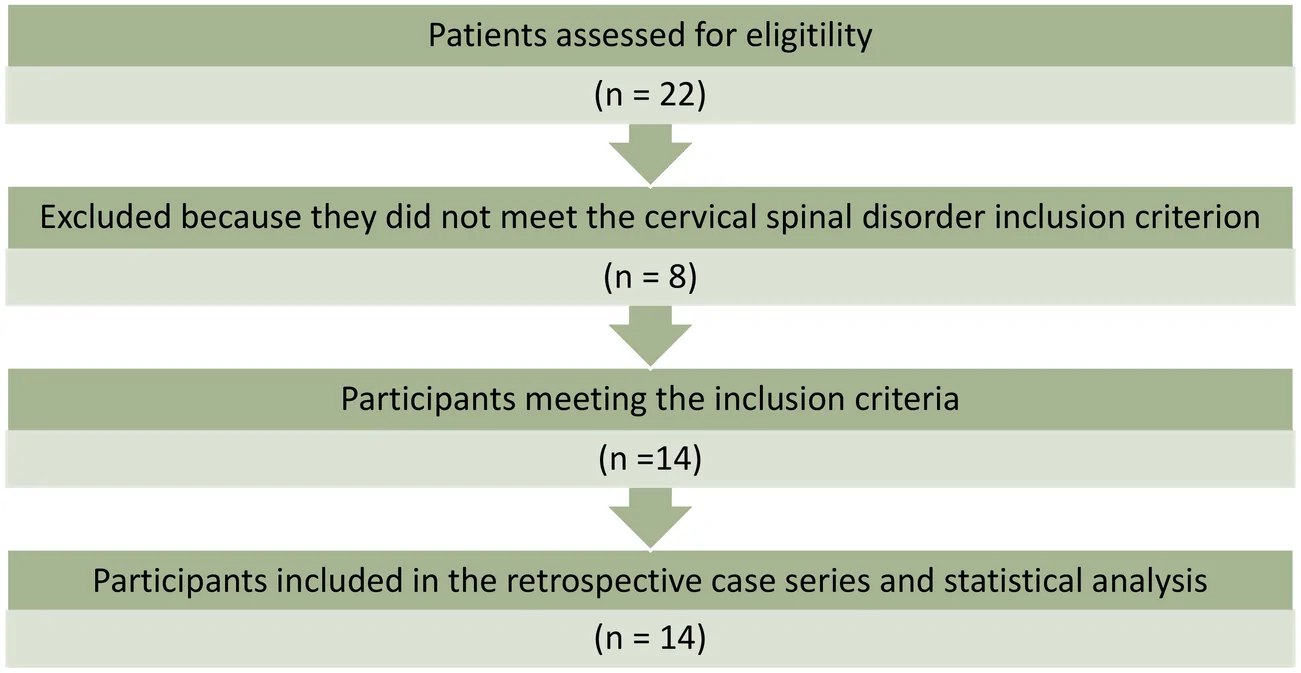
Participant flow diagram.

### Sociodemographic variables

- The study included 14 participants, of whom four were female (28.6%) and 10 were male (71.4%);
- Regarding age, participants ranged from 30 to 79 years, with a mean of 56.2 years. The age group with the highest percentage (42.9%) was 50–59 years (*n* = 6);
- All participants had a diagnosis of cervical pathology; however, the most frequent diagnoses were disc herniation and fracture (*n* = 4 each), followed by space-occupying lesion (*n* = 3), spinal cord trauma without fracture (*n* = 2), and myelopathy (*n* = 1);
- The length of hospital stay ranged from 4 to 106 days, with a mean of 41.4 days.

### Clinical variables

The variable “presence of sensitivity in the affected upper extremity” was assessed prior to the sensorimotor intervention. Of the 14 participants, 8 (57.1%) had no documented tactile sensation in the affected upper extremity, whereas 6 (42.9%) had documented tactile perception. During and following manipulation of the viscoelastic product, all participants (100%) reported perceiving a cooling sensation. This response represented perception of a specific sensory characteristic of the material and was not interpreted as evidence of recovery of baseline tactile sensation.

Regarding spasticity, which was assessed at baseline and at discharge using the Modified Ashworth Scale, dispersion of participants across the recorded spasticity categories was observed at the start of the program, with Grade 1 being the most common (28.6%). At discharge, the majority (71.4%) presented with Grade 0 spasticity (Table 1). A statistically significant change was observed between baseline and discharge (Wilcoxon signed-rank test: *Z* = −3.093, *p* < .001), with a large effect size (*r* = 0.83). The Hodges–Lehmann estimate of the median difference was −1.50 (95% CI: −2.00 to −1.00).

**Table 1:** Distribution of the spasticity level variable (*n* = 14).

Degree of spasticity	Start of the rehabilitation nursing program		Discharge	
	*n*	%	*n*	%
0	1	7,1	10	71,4
1	4	28,6	4	28,6
1+	3	21,4	0	0
2	2	14,3	0	0
3	3	21,4	0	0
4	1	7,1	0	0
Total	14	100	14	100

Grade 1+ was retained as a distinct Modified Ashworth Scale category in the analysis.

Muscle strength was assessed using the MRC scale. Eight participants had unilateral reduced upper extremity mobility and six had bilateral reduced mobility, corresponding to a total of 20 affected upper extremities among the 14 participants. For descriptive purposes, the distribution of MRC grades across all 20 affected upper extremities is presented in Table 2. These limb-level data were used to describe the observed distribution of muscle strength and were not treated as 20 independent observations in the inferential analysis.

**Table 2:** Distribution of muscle strength across the different segments of the affected upper extremities (*n* = 20 upper extremities from 14 participants).

Degree	Fingers		Wrist		Forearm		Elbow		Shoulder	
	Start of the program	Discharge	Start of the program	Discharge	Start of the program	Discharge	Start of the program	Discharge	Start of the program	Discharge
	*n* (%)									
0	—	—	—	—	—	—	—	—	—	—
1	4 (20)	—	4 (20)	—	—	—	—	—	—	—
2	8 (40)	—	4 (20)	—	6 (30)	—	4 (20)	—	3 (15)	—
3	6 (30)	2 (10)	5 (25)	1 (5)	5 (25)	1 (5)	6 (30)	—	5 (25)	—
4	2 (10)	16 (80)	7 (35)	13 (65)	6 (30)	8 (40)	6 (30)	5 (25)	3 (15)	3 (15)
5	—	2 (10)	—	6 (30)	3 (15)	11 (55)	4 (20)	15 (75)	9 (45)	17 (85)
Total	20 (100%)	20	20	20	20	20	20	20	20	20
		100%	100%	100%	100%	100%	100%	100%	100%	100%

Descriptively, the distribution of recorded MRC grades shifted from predominantly lower grades (1–2) at baseline towards higher grades (4–5) at discharge.

The self-care variable, assessed using the Barthel Index, is shown in Figure 2. At baseline, most participants were classified as totally or severely dependent. At discharge, the distribution shifted towards moderate or slight dependence, with two participants classified as totally independent. A statistically significant change was observed in functional independence between baseline and discharge, as assessed using the Barthel Index (*Z* = −3.297, *p* < .001, *r* = 0.88).

**Figure 2:**
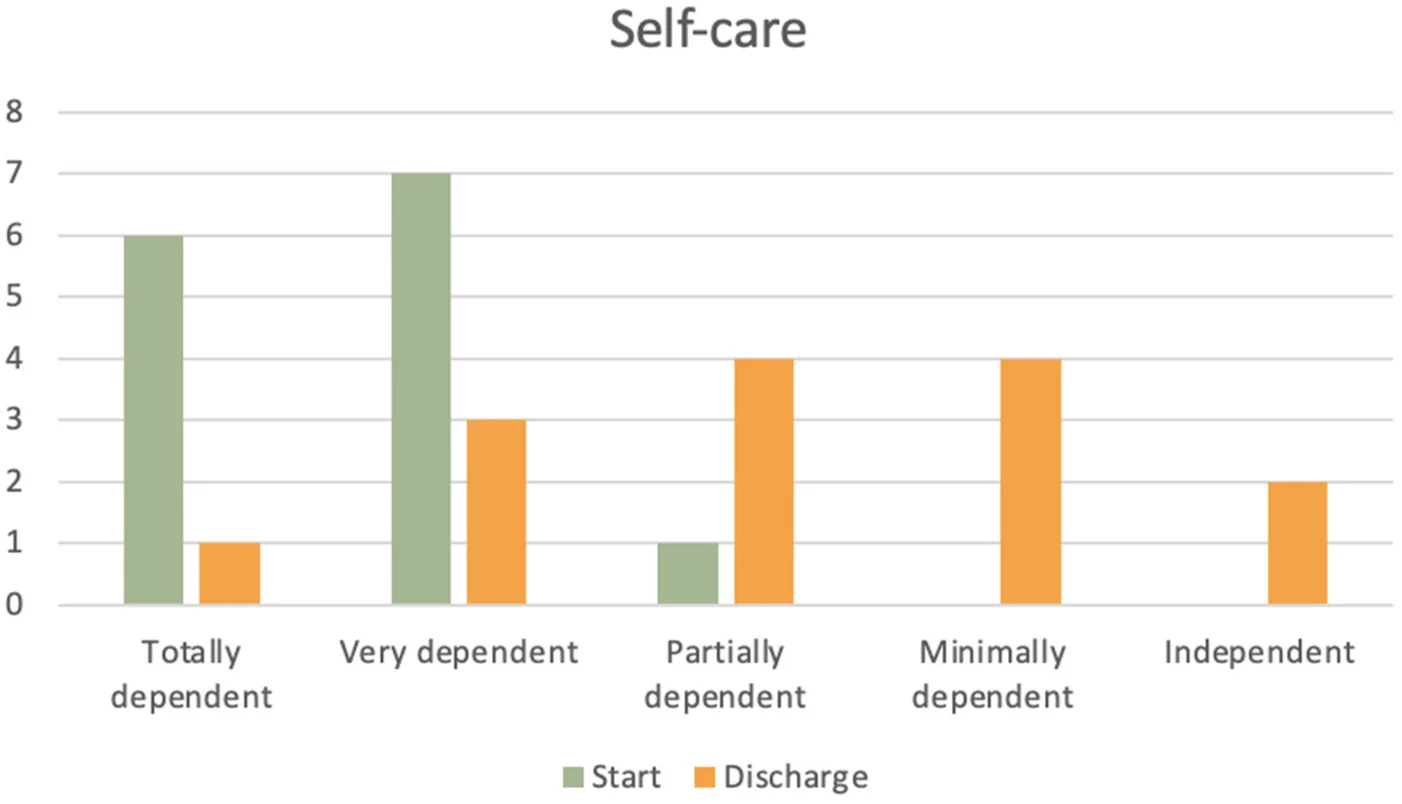
Results for the self-care variable assessed using the Barthel index (*n* = 14).

At the participant level (*n* = 14), Wilcoxon signed-rank tests were used to compare baseline and discharge measurements. Exact two-tailed *p*-values were reported. Statistically significant changes were observed in spasticity (*Z* = −3.093, exact *p* < .001, *r* = .83), finger strength (*Z* = −3.330, exact *p* < .001, *r* = .89), wrist strength (*Z* = −3.324, exact *p* < .001, *r* = .89), forearm strength (*Z* = −2.953, exact *p* < .001, *r* = .79), elbow strength (*Z* = −3.084, exact *p* < .001, *r* = .82), shoulder strength (*Z* = −2.555, exact *p* = .008, *r* = .68), and functional independence measured by the Barthel Index (*Z* = −3.297, exact *p* < .001, *r* = .88) (Table 3).

**Table 3:** Results of the Wilcoxon signed-rank test comparing baseline and discharge measurements (*n* = 14).

Test statistics^a^	Spasticity (final − baseline)	Barthel Index (final − baseline)	Fingers (final − baseline)	Wrist (final − baseline)	Forearm (final − baseline)	Elbow (final − baseline)	Shoulder (final − baseline)
Z	−3.093^b^	−3.297^c^	−3.330^c^	−3.324^c^	−2.953^c^	−3.084^c^	−2.555^c^
Asymp. Sig. (2-tailed)	.002	<.001	<.001	<.001	.003	.002	.011
Exact Sig. (2-tailed)	<.001	<.001	<.001	<.001	<.001	<.001	.008

Exact two-tailed *p*-values are reported. Effect sizes (r) were calculated from the Wilcoxon signed-rank test statistic.

a Wilcoxon Signed Ranks Test.

b Based on positive ranks.

c Based on negative ranks.

### Contextual variables

The number of rehabilitation nursing interventions involving the use of the viscoelastic product ranged from three to 21 sessions per participant, with an average of 8.9 sessions per participant. The documented number of sessions reflects rehabilitation nursing records and does not include unsupervised sessions performed independently or with family assistance. Therefore, the total intervention exposure could not be determined retrospectively.

Regarding the variable “sensory-motor intervention performance,” all participants performed exercises with the viscoelastic product outside supervised rehabilitation nursing sessions; 11 participants performed the exercises independently, while in five cases, family members provided assistance.

Regarding the variable “destination after hospital discharge,” 7 participants (50.0%) were discharged home, 3 (21.4%) were transferred to another hospital, 3 (21.4%) were admitted to the Alcoitão Rehabilitation Medicine Center and 1 (7.1%) was admitted to the National Network of Integrated Continuing Care.

## Discussion

The results of this study showed changes between baseline and discharge during the rehabilitation period in which a sensorimotor intervention using a viscoelastic product was implemented, integrated into a rehabilitation program in the context of surgical hospitalization, particularly in terms of spasticity, muscle strength in the affected upper extremity, and self-care. However, given the retrospective case-series design and the absence of a control or comparison group, these changes cannot be attributed specifically to the intervention, as spontaneous neurological recovery, surgical treatment, standard rehabilitation and the duration of hospitalization may also have contributed to the observed changes. Furthermore, the sample included heterogeneous cervical conditions, including disc herniation, fracture, space-occupying lesion, spinal cord trauma and myelopathy. These conditions differ in pathophysiology, neurological involvement and expected recovery trajectories, which limits the interpretability of pooled changes and precludes assuming a common recovery pattern across participants. Subgroup analysis according to diagnosis was considered but was not feasible because of the small number of participants within each diagnostic category. The following analysis compares these findings with the available evidence, acknowledging from the outset that the specific literature on the application of viscoelastic products in cervical pathology including disc herniation, fracture, and trauma remains scarce supporting the exploratory nature of the present study.

### Spasticity

A statistically significant change in spasticity was observed between baseline and discharge, with most participants (71.4%) exhibiting Grade 0 spasticity at discharge. This finding should be interpreted as a change observed during the rehabilitation period rather than as evidence of a specific treatment effect.

These results are consistent with literature suggesting that tactile sensory stimulation may influence neurophysiological and functional responses during neurorehabilitation (24). In a quasi-randomized clinical trial, the authors found that multisensory stimulation, including gentle tactile stimulation, produced significant effects on neurophysiological and functional measures in patients in a minimally conscious state, supporting the incorporation of tactile interventions into neurorehabilitation programs (24). However, this evidence comes from a clinically different population and should therefore be considered indirect evidence in relation to the participants in the present study.

From a broader cervical spinal cord injury rehabilitation perspective, sensory and task-specific training have also been investigated as strategies to facilitate motor recovery and upper limb function. Systematic reviews have reported improvements in upper limb strength, arm and hand function and activities of daily living following exercise based rehabilitation after cervical spinal cord injury, although the evidence remains heterogeneous and methodological limitations are common (25, 26). These findings provide contextual evidence for the use of sensorimotor and task-specific approaches in cervical neurological rehabilitation, but they do not establish an effect of the viscoelastic product used in the present study.

The sensory characteristics of the viscoelastic product, including its viscosity, elasticity and cooling sensation, may provide tactile and resistive input during upper-limb exercises. However, the present study did not directly measure muscle relaxation, reflex excitability, or neurophysiological responses. Therefore, specific mechanisms linking these sensory characteristics to the observed changes in spasticity cannot be established from the present data. The proposed contribution of peripheral sensory stimulation to motor control therefore remains hypothetical in this context.

Evidence from rehabilitation programs following cervical surgery also suggests that sensorimotor-oriented interventions implemented during hospitalization may be associated with functional gains (27, 28). However, these studies evaluated broader rehabilitation interventions and therefore cannot be used to isolate the contribution of the viscoelastic product to the changes observed in the present case series.

### Muscle strength

Statistically significant differences were observed in all segments of the upper extremity evaluated, with a reduction in the frequency of the lowest grades (1–2) and an overall shift towards higher MRC grades at discharge. These findings indicate changes in recorded muscle strength between baseline and discharge.

These findings are broadly consistent with evidence supporting exercise based and task specific upper limb rehabilitation after cervical spinal cord injury. Previous systematic reviews have reported changes in muscle strength and upper limb motor and functional outcomes following exercise-based interventions, although the available studies vary considerably in intervention type, participant characteristics and methodological quality (26, 29).

This is consistent with previous observations reporting changes in muscle strength and functional outcomes following the use of a viscoelastic product in rehabilitation settings (5, 6). Greater changes in MRC grades were observed in some proximal segments (shoulder and elbow), although the descriptive distribution of MRC grades should be interpreted cautiously given the small sample and the inclusion of both unilateral and bilateral upper-extremity involvement (7, 19). This proximal predominance is also described in the literature on motor recovery following cervical spinal cord injury, in which improvements in proximal strength may precede gains in distal grip strength and dexterity, reflecting the hierarchical organization of neurological recovery pathways (27). In cervical spine conditions with spinal cord involvement, intensive inpatient rehabilitation is associated with clinically significant improvements in upper extremity function, as assessed by standardized motor scales (28). Nevertheless, the findings derive from broader rehabilitation interventions and cannot be directly attributed to the viscoelastic product.

### Functional independence

The results of the Barthel Index revealed a shift from total and severe dependence at baseline towards moderate or slight dependence at discharge, with two participants becoming independent at the time of discharge. These findings are consistent with the relationship described in the literature between upper extremity function and greater independence in self-care (5).

Changes in upper extremity strength and spasticity may be relevant to activities requiring upper limb function, including eating with adapted utensils, personal hygiene, dressing, and participating in transfers (6, 30). In individuals with cervical spine conditions involving spinal cord involvement, improvements in proximal strength and coordination have been associated with gains in functional independence, as reflected by measures such as the Barthel Index (27). However, the present study did not examine whether changes in spasticity or muscle strength directly mediated changes in Barthel Index scores.

The observed change in functional independence should therefore be interpreted within the broader context of acute hospitalization and multidisciplinary rehabilitation. Functional independence during acute hospitalization is influenced by multiple factors, including neurological recovery, surgical management, nursing care, conventional rehabilitation and changes in mobility and self-care capacity over time.

### Contextual variables and continuity of care

Most participants (71.4%) received up to 10 sessions with the Rehabilitation Nurse Specialist, reflecting variability in the number of documented rehabilitation nursing sessions between participants and differences in length of hospital stay (average of 41 days). The number of documented sessions does not necessarily represent the total exposure to the intervention, as participants also performed exercises independently and/or with family support outside formal rehabilitation sessions. Consequently, the documented number of supervised sessions should not be interpreted as the total intervention dose.

Importantly, all participants performed exercises with the viscoelastic product independently and/or with family support, allowing the intervention to be continued outside of formal rehabilitation sessions. This aspect may have increased opportunities for practice, but the retrospective records did not allow the amount or frequency of unsupervised practice to be quantificed. After discharge, 50% of the participants returned home, providing an opportunity for continued participation in daily activities and the application of acquired functional abilities at home.

This transition to the home may also be understood in light of Meleis's Theory of Transitions, which recognizes the active role of the individual and the family in adapting to changes in health status and in developing new knowledge and skills to manage the transition (17). In the present study, this theoretical framework provides a context for understanding continuity of care and the involvement of patients and families, rather than evidence of an effect of the intervention on post-discharge adaptation.

### Gaps in the literature and the study's contribution

It is important to note that the published evidence specifically addressing slime-type viscoelastic products in the rehabilitation of cervical conditions remains very limited, represents both a limitation in terms of the ability to compare findings with previous research and an area of emerging research interest addressed by the present study (5, 7). In contrast, the broader literature on upper-limb rehabilitation after cervical spinal cord injury supports the potential value of repetitive, task-specific and sensorimotor-oriented practice for upper limb motor and functional outcomes, while also highlighting the need for adequately controlled and standardized studies (26, 31). The use of an accessible, low-cost product that can be used independently by the patient and family, and that provides tactile, proprioceptive and resistive sensory input during upper extremity exercises, may be feasible within rehabilitation practice and warrants further investigation. However, feasibility and potential clinical effects should be evaluated prospectively using standardized intervention protocols and appropriate comparison groups.

The findings of this study provide preliminary clinical observations of changes occurring between baseline and discharge that may inform future research using more rigorous methodological designs, namely prospective cohort studies or controlled clinical trials.

### Limitations

This study has several limitations that should be considered when interpreting the findings. First, the small sample size (*n* = 14) and retrospective case-series design limit the generalizability of the findings. No *a priori* power analysis was performed, and the small sample size limits the statistical power of the study and the precision of the estimates. In addition, the absence of a control or comparison group and the before-and-after design make the findings susceptible to regression to the mean and other temporal effects.

Second, the absence of a control or comparison group prevents differentiation between changes potentially associated with the sensorimotor intervention and those related to spontaneous neurological recovery, surgical treatment, standard rehabilitation care, changes occurring during hospitalization. In addition, the absence of a sham or placebo viscoelastic product control prevents differentiation between potential specific effects of the material and non-specific effects, such as attention, therapeutic interaction, participant expectations or increased engagement during rehabilitation.

Third, intervention exposure varied between participants, and some exercises were performed independently or with family assistance, limiting the ability to quantify adherence and standardize the intervention dose. The total intervention dose could not be determined retrospectively because unsupervised practice was not systematically quantificed. The substantial variability in length of hospital stay (4–106 days) and number of documented intervention sessions (3–21) further limits the comparability of intervention exposure between participants and may have resulted in different opportunities for neurological recovery and rehabilitation across participants.

Fourth, the retrospective use of clinical records may have introduced measurement and documentation bias, particularly for sensory findings and intervention exposure. The heterogeneity of the underlying diagnoses, with different pathophysiological mechanisms and expected recovery trajectories, further limits the interpretability of pooled results and represents an important source of clinical confounding. Subgroup analyses by diagnosis were not feasible because of the small number of participants within each diagnostic category.

Fifth, the analysis of muscle strength required consideration of bilateral involvement. Although 20 affected upper extremities were included in the descriptive analysis, inferential analyses were conducted at the participant level (*n* = 14) to avoid treating both upper extremities of the same participant as independent observations. This approach limits the extent to which limb-level changes can be generalized and requires cautious interpretation of the aggregated participant-level MRC data.

Finally, the use of clinical assessment scales and the absence of blinded outcome assessment may have introduced potential measurement bias. The possibility of assessor-related bias cannot therefore be excluded. The number of statistical comparisons performed may also have increased the risk of type I error and no adjustment for multiple comparisons was applied. In addition, no long-term follow-up was performed after hospital discharge, and no neurophysiological or imaging measures were available to directly evaluate the mechanisms underlying the observed changes.

## Conclusion

Overall, the results of this study showed changes between baseline and discharge in upper extremity function among individuals with non-degenerative cervical pathology who participated in a rehabilitation program incorporating a sensorimotor intervention using a viscoelastic product. These changes included changes in spasticity and muscle strength, as well as changes in functional independence and self-care. Given the exploratory nature of this retrospective case series, the observed changes should not be interpreted as evidence of treatment efficacy or attributed specifically to the sensorimotor intervention.

The observed findings provide preliminary clinical observations regarding the use of a viscoelastic product as part of a sensorimotor rehabilitation intervention. Further studies with larger samples, prospective designs, standardized intervention protocols, appropriate control or comparison groups, blinded outcome assessment, clearly defined intervention doses and long-term follow-up are needed to evaluate the effects of this approach and investigate its potential clinical mechanisms.

## Data Availability

The datasets presented in this article are not readily available due to privacy concerns. Requests to access the datasets should be directed to the corresponding author.
